# Future weather dataset for fourteen UK sites

**DOI:** 10.1016/j.dib.2016.07.057

**Published:** 2016-08-03

**Authors:** Chunde Liu

**Affiliations:** Centre for Energy and the Design of Environments, Department of Architecture and Civil Engineering, University of Bath, UK

**Keywords:** Future weather files, Climate change, Overheating risk assessment, Thermal comfort, Heat stress

## Abstract

This Future weather dataset is used for assessing the risk of overheating and thermal discomfort or heat stress in the free running buildings. The weather files are in the format of .epw which can be used in the building simulation packages such as EnergyPlus, DesignBuilder, IES, etc.

**Specifications Table**

**Table:** 

Subject area	*Building science*
More specific subject area	*Thermal comfort*
Type of data	*Figure, EPW files*
How data was acquired	*UKCP09 weather generator*
Data format	*Raw*
Experimental factors	*Select hourly data for fourteen UK sites*
Experimental features	*Future weather files for assessing overheating risk and thermal discomfort or heat stress*
Data source location	*Belfast, Birmingham, Cardiff, Edinburgh, Glasgow, London (Heathrow), Leeds, Manchester, Newcastle, Norwich, Nottingham, Plymouth, Southampton and Swindon.*
Data accessibility	Data is with this article and available via http://doi.org/10.15125/BATH-00190

**Value of the data**

• The dataset could be used for assessing the risk of overheating and thermal comfort or heat stress under a changing climate.
• Building practitioners could use this data to test the building against changing climate to avoid heat-related disaster.
• This dataset could be useful for researchers who are interested in passive design.

## 1 Data

This dataset consists of future weather files for fourteen UK locations. The dataset is created for dynamic thermal comfort simulations taking account of climate change. The future weather files are in the format of .EPW which are commonly used in building simulation packages such as EnergyPlus and DesignBuilder.

## 2 Experimental design, materials and methods

**Fig. 1:**
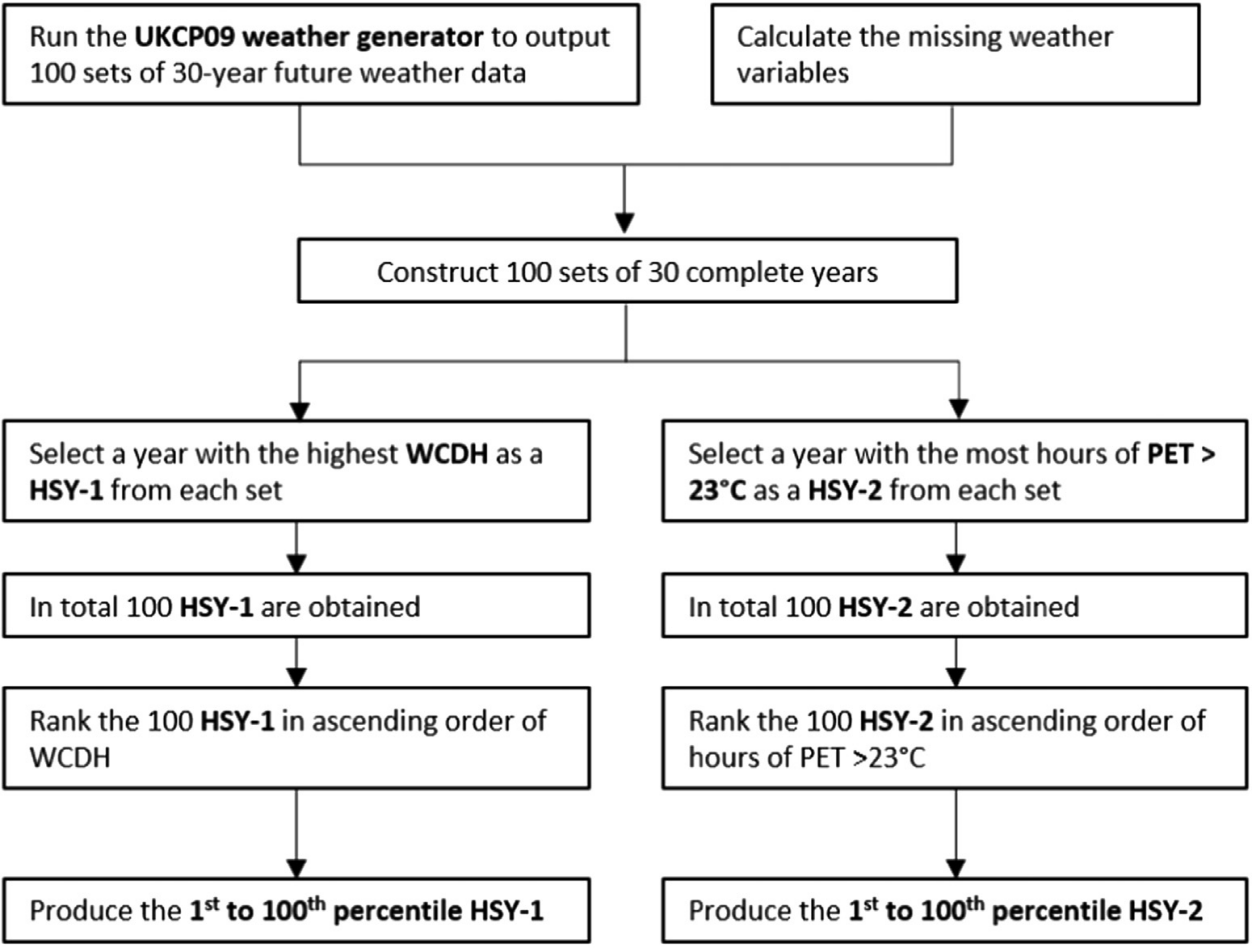
Procedure for creating pHSY-1 and pHSY-2.

The “future hot summer years” are created using the output from UKCP09 weather generator [2]. UKCP09 weather generator offers future weather time series. The outputs incorporates the UKCP09 climate projections. The “future hot summer years” are selected based on the weighted cooling degree hours and physiologically equivalent temperature. They can represent hot summer years to be used in assessing the risk of overheating and thermal discomfort or heat stress. The procedure of the creation is as follows (Fig. 1).

The detailed method is presented in [1].
