# Supplementary material for: Future weather dataset for fourteen UK sites
Source: Data Brief. 2016 Aug 3;8:1308–10. doi: 10.1016/j.dib.2016.07.057 (PMC4990658; doi:10.1016/j.dib.2016.07.057)
Supplement: Supplementary file 1 — Supplementary material [file mmc1.doc]

**Conflicts of Interest**

**Section 1. Identifying Information**

Given Name (First Name): Chunde

Surname (Last Name): Liu

Date: 25 July 2016

Are you the corresponding author? Yes

Manuscript title: Future weather dataset for fourteen UK sites

Manuscript Identifying Number: DIB-D-16-00410

**Section 2. The Work Under Consideration for Publication**

Did you or your institution at any time receive payment or services from a third party (government, commercial, private foundation, etc.) for any aspect of the submitted work (including but not limited to grants, data monitoring board, study design, manuscript preparation, statistical analysis, etc.)? No

Are there any relevant conflicts of interest? No

**Section 3. Relevant financial activities outside the submitted work**

Did you have financial relationship with any organisation or government? No

Are there any relevant conflicts of interest? No

**Section 4. Intellectual Property – Patents & Copyrights**

Do you have any patents, whether planned, pending or issued, broadly relevant to the work? No

**Section 5. Relationship not covered above**

Are there other relationships or activities that readers could perceive to have influenced, or that ive the appearance of potentially influencing, what you wrote in the submitted work?

No other relationships/conditions/circumstances that present a potential conflict of interest.
